# Lectures on Diseases of the Skin

**Published:** 1886-05

**Authors:** Henry Wile

**Affiliations:** Lecturer on Dermatology, Atlanta, Ga.


### LECTURES ON DISEASES OF THE SKIN.

DELIVERED AT THE ATLANTA MEDICAL COLLEGE DURING THE SESSION OF 1885–6. BY HENRY WILE, M. D., LECTURER ON DERMATOLOGY, ATLANTA, GA.

### LECTURE III.

GENERAL SYMPTOMATOLOGY—SUBJECTIVE SYMPTOMS—OBJECTIVE SYMPTOMS, PRIMARY AND SECONDARY—GENERAL CHARACTERISTIC FEATURES.

Reported Stenographically for THE ATLANTA MEDICAL AND SURGICAL JOURNAL BY W. Kay Tewksbury.

Disease of the skin, like disease of other organs, manifests its presence by means of symptoms, and these may be subjective or objective. Subjective symptoms consist of disturbances of the sensory functions of the skin and are appreciated by the patient. The sensory functions are interfered with, being increased or diminished to varying degrees, or may be altogether obliterated as in certain anæsthetic conditions. The subjective symptoms that are commonly encountered are those of itching, burning, pain or formication. These symptoms, when present, the patient becomes cognizant of, and they may give evidence of their existence in the form of excoriations or scratch-marks which are produced by the patient in efforts to get relief.

Objective symptoms, on the other hand, are local manifestations due to structural changes in the cutaneous tissues, and therefore visible to the eye of the observer. They are, as Kaposi aptly says, "the characters which a diseased process has drawn upon the skin." They vary as to types, intensity and mode of development. These symptoms, also known as elementary lesions, may be primary or secondary. Primary objective symptoms are those which characterize the direct formation and development of cutaneous diseases, or they are the visible, tangible forms in which diseases first appear. Such are the macule, papule, tubercle wheal, vesicle, bleb and pustule.

The macule or spot is a non-elevated abnormal discoloration of the skin, due to the presence of an abnormal amount of blood or pigment, or to a want of one or both. Its color and form are manifold, and in size it may vary from a pin-head to a silver dollar and larger. When due to the presence of an abnormal amount of blood, it constitutes a simple hyperæmia of the skin, as in the *erythemata*. Hyperæmic spots may also be the result of a congeries of blood-vessels, as in *nævus vasculosus*, or ordinary mother-mark. Macules may be due to hemorrhagic infiltrations, as in *purpura* (scurvy), and these differ from those resulting from simple hyperæmia by not disappearing on pressure with the finder, for in the former the cutaneous tissues become stained with the coloring matter of the blood. A want or diminution in the amount of blood may contribute to the occurrence of pale, white spots, as in *maculæ atrophicæ* (macular atrophy.) As a result of an increased deposition of pigment, a variety of processes may occur, giving rise to macules, according to the nature, location of the pigment and the manner of its distribution, *e. g.*, in *lentigo* (freckle) and *chloasma*. Then again, by reason of a want of pigment, partial or entire, there may occur certain colorless conditions of the skin and hair, *e. g.*, *albinism*, *vitiligo*, *canities*.

Macular lesions may be congenital, as in *nævus*, acquired as in *chloasma*, or artificial as a result of tattooing. They may be temporary as in *roseola*, or persistent as in *vitiligo*.

The papule is a pin-head to pea-sized solid elevation of the skin, having a consistence according to the nature of its contents. It is the result of numerous processes, and varies in form and color according to location and composition. It may be due to a retention of sebaceous matter in a sebaceous gland, or its outlet, as in *milium*, or to a circumscribed inflammation of the papillary layer of the corium, as in *papular eczema*, or to a dense, sharply defined cellular infiltration of the corium, as in *papular syphiloderm*. Then again, collections of epidermal tissue, enlargement of the papillæ of the corium, follicular and peri-follicular inflammation, and finally new formations in the tissue of the corium may lead to the formation of the papule.

The tubercle is a lesion having the characteristics of the papule, and presenting almost the same pathological significance, but differing in size, varying from the size of a pea to that of a hazel-nut. It may be deeply seated in the subcutaneous tissue, so that it can only be detected by palpation, but as a rule it projects from the surface.

Wheals are elevated pea to silver dollar sized, flattened, pink or pinkish-white, evanescent efflorescences. They are due to serous exudations into the papillary layer of the corium ann rete, and are characterized by their rapid development, appearing suddenly, it may be in a moment, and having as a rule a short existence, quickly fading away. The lesions of *urticaria* and those resulting from irritation produced by the stings of insects may be cited as examples of wheals. They are always attended with itching or burning sensations, and by rubbing or scratching the lesions are increased in size and become at once more prominent.

The vesicle is a pin-point to pea-sized elevation of the upper layers of the epidermis, resulting from a serous, occasionally bloody exudate, which is usually at first clear, later becoming cloudy. As the exudate accumulates it lifts up the epidermal layers and causes an elevation. The covering of the lesion may be thick or thin, according as the exudation is in the upper or lower layers of the epidermis. and when occurring in different layers of the epidermis, it gives rise to the formation of compartments. The typical vesicle is almost transparent, and in going through degenerative changes it grows opaque. In disappearing, there is generally a depression or umbilication formed in the centre of the lesion. The exudation comes from the papillary blood-vessels and accumulates between the layers of the epidermis, as a rule separating the rete and horny layers. Exudations may also take place into the follicles and ducts of the glands, more especially those of the sweat glands, as in *sudamina*. The vesicle may develop directly, as in the affection just mentioned, or indirectly from the papule, as in some forms of *eczema*, and may disappear by absorption of its contents, or by degeneration with formation of pus, giving rise to the pustule.

The bleb has the same structure as the vesicle, and also possesses its general characteristics, differing only as to size, varying from a pea to an egg in size. It may develop at once from a serous or bloody exudation into the epidermis, or may result from the coalition of two or more neighboring vesicles. This lesion is commonly seen as the result of burns, and occurs also in the disease called *pemphigus*. In the former case the exudate is often deeply seated, and the covering of the lesion is thick; whereas in the latter it is more superficial, having a thin covering which may easily be ruptured.

The pustule differs from the vesicle only in the composition and color of the exudation, that of the former containing pus corpuscles, and being therefore as a rule of a yellowish or greenish hue. The exudation may occur between the layers of the epiderm, as in *impetigo*, in and around the sebaceous glands, as in *acne*, or around the hair follicles, as in *sycosis non-parasitica*. It may begin as a pustule or be a subsequent stage of the vesicle, and in the latter case there may be a middle stage of development, in which the lesion is known as vesico-pustule. The exudation may be superficial, confined to the layers of the epidermis, or may involve the layers of the corium. As suppuration always indicates destruction of tissue, the pustule may or may not be followed by a scar, according to the layers of skin affected by the process. If epidermis only is destroyed, new epidermal cells will be formed without leaving a scar. If the papillary layer of the corium is involved, it is never restored as such, but cicatricial tissue will be formed in the process of reparation and a scar results. This may occur in *variola*, *acne* and *syphilis*. The involution of a pustule may take place by absorption of its contents, without rupturing, or by desiccation after rupture and discharge of its contents on the surface. Before disappearing it may become umbilicated, especially if situated around a hair follicle or glandular duct.

A tumor is a variously sized new formation which may appear on any part of the body, and is generally seated in the lower layers of the skin. It occurs for the most part as the result of chronic inflammation or hypertrophy of one or all the layers of the skin. It may be present in various shapes, having a convex surface or presenting more or less irregularity of surface. It may have a broad base or be attached by a pedicle.

Secondary symptoms always appear as a result of previously existing disease, and are the subsequent stages of development or the residua of the primary forms just described. Under this group are embraced scales, crusts, excoriations, fissures, ulcers, scars and pigmentation.

Scales are collections of desquamated epidermal cells. They may be dry or oily, according to the activity of the cutaneous glands, or they may be mixed with discharges and foreign matter. Scales may be thin or thick, large or small, according to the superficial or deep character of the morbid process. Desquamation takes place physiologically, but is then imperceptible. It becomes pathological when it occurs in visible quantity.

Crusts are masses that result from the desiccation of discharges or exudations on the surface of the skin. The discharges or exudations may be composed of pure serum, pus or blood, or a mixture of these. When composed of serum the crusts are of a yellowish color; when of pus, greenish; when of blood, brownish or black. But a variety of colors may occur as the result of the admixture of foreign matter. They may be in the form of a thin lamella, as in *eczema*, or may be bulky and made up of superimposed layers, as in *syphilitic rupia*.

Excoriations are lesions which result from the removal of cutaneous tissue by mechanical injury. The injury may be accidental, as by coming forcibly in contact with a sharp or rough surface, or it may be self-inflicted, as by scratching with the finger-nail. The most common are those which are the result of scratching, and they form a valuable diagnostic sign, indicating the existence of nerve irritation that gives rise to itching. They exist in various sizes and shapes, but as a rule are linear, and the depth of the lesion or loss of tissue depends upon the duration and intensity of the irritation. The lesion may consist of a removal of the superficial layers of the epidermis, which in a few days is restored, or of the deeper layers, giving rise to a slight serous exudation that dries and forms a crust. Should the irritation be intense, all the layers of the epidermis and the papillary layer of the corium may be torn off, causing a slight bloody exudation that dries and forms a dark crust that is subsequently thrown off, leaving a scar as the result. If the irritation is discontinued the parts return to their normal condition. If, however, the irritation is kept up for a time, the skin becomes congested, coloring matter of the blood escapes into the surrounding tissues, and as a result there is developed a pigmented condition.

Fissures consist of cracks or clefts of the cutaneous structures, and may extend into the corium. These clefts may be sharp in their definition, as in eczema of the fingers, or they may present an indurated border, as in *syphilis*. They generally occur in those regions in which the skin is more or less put upon the stretch, as around the joints of the fingers, the anus and the mouth, and are the result of a previous inflammation, or any process which has a tendency to diminish the elasticity of the skin.

Ulcers are such lesions as result from pathological processes attended with a loss of cutaneous tissue. They are variously sized, and exhibit as a rule little tendency to heal, slowly undergo change, and are inclined to become chronic. The special features to be noted are in regard to the base, the border and the form. The base may be smooth, uneven or covered with hypertrophic granulations. It may exhibit irregular depressions and elevations or papillary excrescences. The base may yield a scanty or copious, thick or thin, watery or bloody, serous or purulent secretion which, according to the amount discharged, may on drying give rise to the formation of variously sized crusts. The border of the ulcer may be smooth, rough, elevated, flattened, ragged, undermined, indurated or non-indurated ; and as regards the form, it may be round or crater-like, kidney-shaped or serpiginous. In the event of healing, a scar always results.

A cicatrix or scar is a new formation of tissue which takes the place of tissue lost by any destructive process involving the connective tissue layers of the skin. The form and size of it is like the lesion it follows; and as to color, it is at first pink or pinkish-white, later growing pale, becoming white on account of disappearance of the blood-vessels. It may be elevated or depressed, smooth or uneven, soft or resistant, and never contains any dermal appendages. It has a tendency to contract, as in burns, and is permanent.

Pigmentation is a discoloration due to the deposition of coloring matter under certain pathological conditions. It may be temporary or permanent, and at times is a symptom of some value in diagnosis.

There are, in addition to the symptoms just delineated, certain features that are in a measure characteristic of the eruptions they accompany, and it is important to keep them in mind when studying a given cutaneous disease. An eruption may be made up of any number of primary and secondary symptoms, and where several are present it is called polymorphous. The symptoms occur in varying degrees of intensity, according to the nature of the process. As to distribution, the eruption may be universal, as in most of the acute *exanthemata;* local, as in the later stages of *syphilis;* symmetrical, as in the early stages of *syphilis;* asymmetrical, as in *epithelioma.*

The localization is sometimes peculiar, as the face is the favorite seat for *lupus erythematosus*, the back of the elbows and surface over the knees for *psoriasis* and *ichthyosis.* Then, again, the eruptions of early *syphilis* and *scabies* have a predilection for the flexor surfaces; whereas *eczema*, as a rule, appears on extensor surfaces. There are but a few examples of localization. Then, again, the lesions composing the eruption may present a peculiar arrangement or configuration, being crescentic, as in some of the syphiloderms, or circular, as in *tinea circinata* (ringworm) and *herpes iris*. The lesion may also be arranged in parallel lines, either straight or curved. This configuration is often determined by the anatomy of the skin, the natural lines or furrows, not infrequently by the course of the nerves, as is shown in *herpes zoster*, and doubtless by the arrangement of the blood-vessel system, the hair follicles and glandular apparatus. These arrangements of lesions are called according to kind, as crescentic, circinate, gyrate, annular, marginate ; and according to other circumstances eruptions are variously characterized, as senilis, infantum, acutus, chronicus, hiemalis, capitis, facialis, etc., etc.

